# Disorganization of Oscillatory Activity in Animal Models of Schizophrenia

**DOI:** 10.3389/fncir.2021.741767

**Published:** 2021-10-05

**Authors:** Lucinda J. Speers, David K. Bilkey

**Affiliations:** Department of Psychology, Otago University, Dunedin, New Zealand

**Keywords:** oscillations, schizophrenia, hippocampus, prefrontal cortex, synchrony, theta, gamma, phase precession

## Abstract

Schizophrenia is a chronic, debilitating disorder with diverse symptomatology, including disorganized cognition and behavior. Despite considerable research effort, we have only a limited understanding of the underlying brain dysfunction. In this article, we review the potential role of oscillatory circuits in the disorder with a particular focus on the hippocampus, a region that encodes sequential information across time and space, as well as the frontal cortex. Several mechanistic explanations of schizophrenia propose that a loss of oscillatory synchrony between and within these brain regions may underlie some of the symptoms of the disorder. We describe how these oscillations are affected in several animal models of schizophrenia, including models of genetic risk, maternal immune activation (MIA) models, and models of NMDA receptor hypofunction. We then critically discuss the evidence for disorganized oscillatory activity in these models, with a focus on gamma, sharp wave ripple, and theta activity, including the role of cross-frequency coupling as a synchronizing mechanism. Finally, we focus on phase precession, which is an oscillatory phenomenon whereby individual hippocampal place cells systematically advance their firing phase against the background theta oscillation. Phase precession is important because it allows sequential experience to be compressed into a single 120 ms theta cycle (known as a ‘theta sequence’). This time window is appropriate for the induction of synaptic plasticity. We describe how disruption of phase precession could disorganize sequential processing, and thereby disrupt the ordered storage of information. A similar dysfunction in schizophrenia may contribute to cognitive symptoms, including deficits in episodic memory, working memory, and future planning.

## Introduction

Schizophrenia is a complex neurological disorder that affects approximately one percent of the population worldwide (Jablensky, 2000; McGrath et al., 2008), and is a leading contributor of the global disease burden (Lopez et al., 2006). It is characterized by a heterogenous constellation of aetiological risk factors, pathophysiological mechanisms, and symptoms. These include positive symptoms, such as hallucinations and delusions, negative symptoms, such as flattened affect and avolition, and broad cognitive disturbances including episodic and working memory, attention, and executive function (Insel, 2010; Barch and Ceaser, 2012; Fusar-Poli et al., 2012; Cannon, 2015). Although the positive and negative symptoms of the disorder have historically received more attention, a growing number of studies investigating cognitive dysfunction in schizophrenia have provided evidence that these impairments are not only a critical factor in predicting poor functional outcomes (Green, 1996), but that they also precede the onset of positive symptoms by almost a decade (Kahn and Keefe, 2013). These findings have prompted some to argue that schizophrenia should be recognized as primarily a cognitive disorder and that the development of new diagnostic tools and treatments has been hampered by the continued focus on psychotic symptoms at the expense of the underlying cognitive disturbances that generally precede them (Elvevag and Goldberg, 2000; Lesh et al., 2011; Kahn and Keefe, 2013).

One feature of schizophrenia is an inability to organize the elements of cognition into a cohesive whole (Javitt, 2009; Fornito and Bullmore, 2015; Friston et al., 2016). In line with this proposal, a growing number of studies have begun to focus on the disorganization of cognitive processes (König et al., 2001; Olypher et al., 2006; Minor and Lysaker, 2014). In particular, complex cognitive operations such as episodic memory and executive function require the dynamic integration of diverse information streams, including both top-down information about beliefs and expectations based on prior experience, as well as lower-level sensory, emotional, and motor information (Engel et al., 2001; Jardri and Denève, 2013). How distributed networks manage the appropriate integration, segregation and sequential ordering of such information remains an open question, although it has become increasingly clear that phase coding mechanisms, in which the temporal spiking of single cells is organized relative to synchronous oscillatory activity occurring at the network level, is likely to play a critical role (Gray et al., 1989; Lisman and Buzsáki, 2008; Buzsáki, 2010).

There is now a large body of literature demonstrating that disturbed oscillatory activity in schizophrenia is often correlated with broad cognitive impairments (Spencer et al., 2004; Schmiedt et al., 2005; Cho et al., 2006; Light et al., 2006; Basar-Eroglu et al., 2007; Haenschel et al., 2009; Uhlhaas and Singer, 2010; Kirihara et al., 2012; Senkowski and Gallinat, 2015; Barr et al., 2017; Adams et al., 2020). Post-mortem studies from individuals with schizophrenia have also provided vital information about basic-level disturbances that occur in schizophrenia, including specific disruptions at the site of N-methyl-D-aspartate (NMDA) receptors (Catts et al., 2016), as well as several GABA disturbances, particularly in regards to glutamic acid decarboxylase 67 (GAD67) and parvalbumin (PV+) expression (Akbarian and Huang, 2006; Fung et al., 2010; Gonzalez-Burgos et al., 2015; Kaar et al., 2019). These findings have led to promising hypotheses that schizophrenia may result from an imbalance of excitation/inhibition in key regions associated with schizophrenia pathology, including the prefrontal cortex (PFC) and the hippocampus (Lewis et al., 2005; Uhlhaas, 2013; Starc et al., 2017). However, direct evidence of how the structural, cellular, and molecular disturbances that are frequently observed in schizophrenia are causally linked to cognitive dysfunction has been more difficult to obtain (Wright et al., 2000; Heckers and Konradi, 2002; Harrison, 2004; Moghaddam and Javitt, 2012; Haijma et al., 2013; Van Den Heuvel and Fornito, 2014; Forsyth and Lewis, 2017). This is known as the problem of the “missing middle,” in which the mesoscopic network processes that bridge the gap between microscopic disturbances and macroscopic behavioral outcomes have remained relatively opaque (Laughlin et al., 2000; Kao et al., 2017).

Bridging this gap is difficult with human subjects, as current non-invasive imaging tools do not provide adequate resolution to determine how basic level disturbances occurring at the cellular level manifest into disorganized network activity and consequent cognitive impairments. The refocusing of research on cognitive disturbances has thus provided an important opening for research involving animal models of schizophrenia, as cognitive disturbances can be more readily measured in animals, unlike the more subjective symptoms of psychosis. Animal models of schizophrenia also provide better access to biological and network mechanisms, as well as providing the opportunity for more targeted manipulations. Such models are, therefore, likely to provide a crucial step in bridging the missing “middle,” as well as providing important information about both primary etiological causes and developmental trajectories.

This review will critically outline the current state of studies that have investigated disorganized oscillatory activity in animal models of schizophrenia, with a specific focus on the hippocampus. The first section will provide the rationale for investigating disorganized oscillatory activity in schizophrenia, as well as a brief overview of the findings and limitations of such studies in humans (for a more detailed review of disturbed oscillatory activity in individuals with schizophrenia, readers are referred to the review by Uhlhaas and Singer, 2010). The main body of the review will then focus on evidence accumulating from animal models of the disorder, including models of genetic risk, maternal immune activation (MIA), and models of NMDA receptor (NMDAR) hypofunction. We will present a critical analysis of these findings in relation to gamma and theta frequency oscillations, sharp-wave ripples (SPW-Rs), and theta phase precession, including the functional implications of disorganized oscillatory activity for cognitive processes that have been associated with these phenomena.

## EEG and MEG Studies in Individuals with Schizophrenia

According to the dysconnection hypothesis, the core symptoms of schizophrenia proceed from the functional disintegration of specialized systems within the brain, including both the intrinsic connections within a local cell assembly and long-range connectivity between distinct brain regions (Friston, 1998; Friston et al., 2016). Robust evidence of functional dysconnectivity in schizophrenia has been provided by a range of non-invasive techniques such as functional magnetic resonance imaging (fMRI), magnetoencephalography (MEG), and electroencephalography (König et al., 2001; Liang et al., 2006; Hinkley et al., 2010; Pettersson-Yeo et al., 2011; Fornito et al., 2012; Di Lorenzo et al., 2015). In particular, MEG and EEG imaging techniques have provided valuable information about the amplitude, frequency, and coherence of rhythmic network activity at high temporal resolutions. These techniques have routinely demonstrated abnormal activity in both schizophrenia patients and their first-degree relatives in the theta (~2–10 Hz), beta (~12–30 Hz), and gamma (~30–90 Hz) frequency bands. These findings suggest that disorganized activity in these bands could be a potential endophenotype of the disorder (Uhlhaas and Singer, 2010; Williams and Boksa, 2010; Moran and Hong, 2011; Kirihara et al., 2012; Berger et al., 2016; Adams et al., 2020). Changes in oscillatory activity may either reflect or underlie a failure of coordinated network synchrony within and across several brain regions, consistent with the proposals that schizophrenia is predominantly a disorder of distributed neural dynamics rather than localized deficits (von der Malsburg et al., 2010; Uhlhaas and Singer, 2015).

Although these previous studies have provided critical evidence that oscillatory activity across several frequency bands is disorganized in schizophrenia, the non-invasive MEG and EEG techniques that are used in these studies are inherently limited in several respects. For example, the spatial resolution of these techniques is relatively low, and despite numerous technological advances that have improved the quality of source localization, the issue of field spread means that precise spatial localization of signal sources must be interpreted cautiously (Schoffelen and Gross, 2009). This issue is particularly important in regards to oscillatory activity that is generated in deeper brain regions, such as the hippocampus, where signals are more prone to distortion. Such issues are not fully resolved using invasive recording techniques, but a comparison of simultaneously obtained invasive and non-invasive EEG recordings in humans has demonstrated that the signal quality of invasive EEG recordings is ~20–100 times better than non-invasive recordings (Ball et al., 2009).

Recent findings in animal models have also demonstrated that the precise temporal spiking of single cells in relation to background local field potential (LFP) oscillations is likely to be functionally important for both low-level plasticity-related processes and for high-level cognition that depends on sequential processing mechanisms (Buzsáki, 2015; Buzsáki and Tingley, 2018; Drieu and Zugaro, 2019). While these synchronizing phenomena appear to occur in humans (Liu et al., 2019; Qasim et al., 2020) they cannot readily be investigated with non-invasive techniques. Thus, although MEG and EEG studies provide important correlational evidence that disturbed network synchrony is likely associated with poor performance across a range of cognitive domains, direct evidence that these phenomena are causally linked is difficult to obtain with these techniques alone. Similar difficulties are apparent in regards to the cellular and molecular basis of oscillatory disorganization. Although a number of basic-level studies have begun to uncover the biological mechanisms of coordinated oscillatory activity (Buzsáki and Draguhn, 2004; Buzsáki and Wang, 2012; Colgin, 2013; Buzsáki, 2015; Drieu and Zugaro, 2019), it remains unclear how the complex aetiological and developmental processes associated with schizophrenia manifest into disorganized oscillatory activity at critical stages of disease progression. Animal models of schizophrenia provide a unique opportunity to resolve some of these issues, and given that the scaling and hierarchical organization of oscillatory activity is evolutionarily preserved across several species (Buzsáki et al., 2013), animal models may be able to provide important translational data across all levels of micro- meso and macroscopic dysfunction.

## Animal Models of Schizophrenia

Over the past few decades, several animal models of schizophrenia-risk have been developed, including genetic, developmental, lesion, and drug-induced models (Jones et al., 2011; Rapoport et al., 2012; Brown and Meyer, 2018; Lee and Zhou, 2019). This diversity reflects the heterogenous range of aetiological factors and pathophysiological mechanisms linked to schizophrenia. The specific disruptions associated with each model provide valuable information about the fundamental biological mechanisms of schizophrenia and allow for investigations of both the acute and longitudinal effects of known risk factors in isolation, and with greater control over the confounding effects of environment and medication. However, these advantages come at a cost, providing a simplified account of schizophrenia pathophysiology that is unlikely to capture the full complexity of the disorder. For example, current evidence suggests that schizophrenia does not emerge from a single genetic, biological or environmental cause, but rather through the complex interplay of these factors, including epigenetic mechanisms that converge on shared pathways of molecular dysfunction (Fatemi and Folsom, 2009; Horváth and Mirnics, 2015). One of the challenges of working with animal models is, therefore, to integrate the findings from these diverse models into a broader understanding of schizophrenia pathology.

Several recent reviews have begun to identify some of the common network disturbances observed in pre-clinical models, although most of these reviews have focused predominantly on the gamma frequency band (Uhlhaas and Singer, 2015), and models of NMDAR hypofunction have been more extensively reviewed than models of genetic and environmental risk factors (Jadi et al., 2016; Cadinu et al., 2018; Krajcovic et al., 2019; Bianciardi and Uhlhaas, 2021). The following section will briefly outline three types of animal models that have been used to investigate network disturbances associated with schizophrenia—models of NMDA hypofunction, genetic risk models, and maternal immune activation (MIA) models, with a focus on how the basic cellular disturbances associated with these models could contribute to the disorganized oscillatory activity.

### NMDAR Hypofunction Models

Considerable evidence points to abnormal glutamate signaling in schizophrenia, particularly at the site of the NMDA subtype of glutamate receptors (Moghaddam and Javitt, 2012; Balu, 2016; Nakazawa and Sapkota, 2020). A transient induction of schizophrenia-like psychosis can also occur in humans following administration of NMDAR antagonists, leading to proposals that changes in glutamate signaling are fundamental to the disorder (Krystal et al., 1994; Umbricht et al., 2000; Moghaddam and Javitt, 2012). Several different animal models of NMDAR dysfunction have thus been developed to determine how NMDAR hypofunction contributes to schizophrenia pathophysiology, including those relying on the acute administration of the antagonist ketamine or MK-801, as well as various NMDAR knockout models that allow researchers to examine the more chronic effects of disturbed NMDAR transmission during early development (Olney et al., 1999; Lee and Zhou, 2019). Since NMDA receptors occur on both principal cells and inhibitory interneurons, a disturbance in these systems has the potential to disrupt the excitatory/inhibitory balance within a network, as well as to modify the oscillatory function that depends on feedback inhibition in order to produce cycles of activity. Theoretically, this could have profound implications for the development and temporal coordination of complex neural circuits, and experimental evidence has confirmed that fast-spiking interneurons, including PV+ cells, are critical for organized oscillatory activity in both the gamma and theta frequency ranges (Cobb et al., 1995; Sohal et al., 2009; Wulff et al., 2009; Stark et al., 2013; Amilhon et al., 2015).

Both acute and chronic NMDA hypofunction have been shown to affect oscillatory activity in NMDAR antagonist models across a range of frequency bands, and these studies are discussed in greater detail in the relevant sections below. Broadly speaking, these studies have provided robust evidence that disrupted NMDAR signaling leads to disturbed oscillatory activity in a number of brain regions (Ma and Leung, 2000; Cunningham et al., 2006; Pinault, 2008; Dzirasa et al., 2009; Hakami et al., 2009; Belforte et al., 2010; Carlén et al., 2012; Kittelberger et al., 2012; Kocsis, 2012; Caixeta et al., 2013; Kalweit et al., 2017; Aguilar et al., 2021). There is also evidence that disturbed oscillatory activity in NMDAR hypofunction models is mediated by abnormal synaptic inhibition, particularly by PV+ interneurons (Carlén et al., 2012; Kittelberger et al., 2012). It remains unclear however whether NMDA hypofunction and other GABAergic disturbances arise independently (Coyle, 2004; Gonzalez-Burgos and Lewis, 2012), although current evidence suggests that the timing of NMDAR manipulations is critical for the development of inhibitory circuits (Wang and Gao, 2009; Belforte et al., 2010). In line with this proposal, one study has demonstrated that the selective deletion of NMDA receptors from predominantly PV+ interneurons during early development triggers several molecular, physiological, and behavioral phenotypes reminiscent of schizophrenia, including spatial working memory impairments, social withdrawal, and reduced pre-pulse inhibition, as well as reduced network synchrony in the somatosensory cortex. The same manipulation had no effect however when performed on post-adolescent mice (Belforte et al., 2010).

### Genetic Risk Models

Although models of NMDAR hypofunction provide important information about how NMDAR signaling contributes to abnormal oscillatory activity, such models may be lacking in ecological validity. Models based on either genetic or environmental risk factors can address this issue to some extent, although the specific biological mechanisms that contribute to abnormal oscillatory activity are more difficult to identify.

Numerous studies indicate that schizophrenia is likely to have a substantial hereditary component (Cardno et al., 1999; Sullivan et al., 2003; Lichtenstein et al., 2009; Harrison, 2015). A number of genomic regions that may confer an increased risk of developing schizophrenia have been identified, although most genetic variants associated with the disorder involve non-coding regions of DNA, indicating that they are predominantly involved in regulating gene expression, such as the timing, abundance, and location of transcription events, rather than encoding for protein sequences themselves (Harrison, 2015; Kahn et al., 2015). Consistent with proposals that schizophrenia is predominantly a neurodevelopmental disorder (Bullmore et al., 1997; Fatemi and Folsom, 2009), several risk variants are also preferentially expressed during fetal development, suggesting that the normal developmental processes of neuronal proliferation, differentiation, and migration may be disrupted during this critical period (Walsh et al., 2008; Birnbaum and Weinberger, 2017).

In particular, genes associated with neuregulin signaling have often been implicated in schizophrenia, and neuregulin is known to play an important role in the development of inhibitory circuits, synaptic plasticity, and axon myelination during critical stages of development (Stefansson et al., 2002; Brinkmann et al., 2008; Mei and Xiong, 2008; Neddens et al., 2011; Ting et al., 2011). Other genes that are involved in early neurodevelopment and maturational processes, such as the Disrupted-in-Schizophrenia 1 (DISC1) gene, appear to exert delayed behavioral and neurochemical effects following pre- and perinatal insults in mice, with measurable effects only appearing after puberty, clearly mirroring the developmental trajectory of schizophrenia in humans (Niwa et al., 2010). Both DISC1 and neuregulin have also been associated with disturbed parvalbumin (PV+) expression in the hippocampus and the PFC (Hikida et al., 2007; Shen et al., 2008; Fazzari et al., 2010), as well as diminished complexity of dendritic spines in hippocampal regions, attenuated synaptic plasticity, and several cognitive phenotypes associated with the disorder (Li et al., 2007; Kvajo et al., 2008; Shamir et al., 2012). Similar neurodevelopmental disturbances have been observed in mouse models of 22q11 microdeletion (Paylor et al., 2001; Mukai et al., 2008, 2015). Taken together, these studies suggest that a range of genetic risk factors disrupt the development of neural circuits, with the most prominent effects emerging after adolescence.

### Maternal Immune Activation (MIA) Models

A number of epidemiological studies indicate that maternal infection during the first and second trimesters is associated with an increased risk of developing schizophrenia in affected offspring (Mednick et al., 1994; Susser et al., 1996; Brown and Derkits, 2010; Selemon and Zecevic, 2015). Subsequent studies have revealed that exposure to proinflammatory cytokines at critical stages of neurodevelopment affects neuronal proliferation and synaptogenesis, which could potentially have profound consequences for the development of neural circuits (Gilmore and Jarskog, 1997; Meyer et al., 2009a, b; Watanabe et al., 2010; Selemon and Zecevic, 2015).

MIA has been extensively modeled in rodents using a variety of induction protocols, including exposure to polyriboinosinic: polyribocytidilic acid (PolyI:C), a synthetic analog of double-stranded RNA that regulates acute responses to viral pathogens (Meyer et al., 2009a, b; Boksa, 2010; Wolff and Bilkey, 2010; Brown and Meyer, 2018; Kentner et al., 2019). The PolyI:C model has been shown to trigger a range of biophysical and molecular abnormalities consistent with schizophrenia, including decreases in hippocampal volume (Zuckerman et al., 2003; Piontkewitz et al., 2011; Crum et al., 2017), altered GAD and PV+ expression (Piontkewitz et al., 2012; Dickerson et al., 2014; Canetta et al., 2016; Cassella et al., 2016; Steullet et al., 2017), reduced inhibition (Zhang and van Praag, 2015), an increased glutamate/GABA ratio in the hippocampus (Patrich et al., 2016), abnormal synaptic plasticity (Savanthrapadian et al., 2013), and dopaminergic dysfunction (Zuckerman et al., 2003; Ozawa et al., 2006; Luchicchi et al., 2016).

A range of behavioral abnormalities that match the symptomatic profile of schizophrenia have also been observed, including several cognitive deficits that have also been associated with disorganized oscillatory activity (Fatemi and Folsom, 2009; Meyer et al., 2009a, b; Brown and Derkits, 2010), These include reduced PPI (Ozawa et al., 2006; Wolff and Bilkey, 2010; Howland et al., 2012; Zhang and van Praag, 2015; Luchicchi et al., 2016), reduced behavioral flexibility (Zuckerman and Weiner, 2005; Bitanihirwe et al., 2010; Savanthrapadian et al., 2013; Ballendine et al., 2015; Kleinmans and Bilkey, 2018), temporal processing disturbances (Deane et al., 2017), and spatial memory impairments (Meyer et al., 2008; Wolff et al., 2011; Murray et al., 2017).

## The Importance of Hippocampal and Prefrontal Oscillations for Cognitive Processes, and Implications for Schizophrenia

Disorganized oscillatory activity has been documented throughout several brain regions in individuals with schizophrenia (Uhlhaas and Singer, 2010), and this current review is not exhaustive. Instead, we have chosen to focus on disorganized activity that occurs in hippocampal and frontal regions in the gamma, theta, and sharp-wave ripple bands. We also discuss how this may influence hippocampal-prefrontal functional connectivity.

Considerable evidence suggests that the temporal coordination of hippocampal activity is critically important for a range of cognitive processes, including episodic, relational, spatial, and working forms of memory, as well as flexible decision making (Buzsáki and Moser, 2013; Colgin, 2016; Drieu and Zugaro, 2019). The laminar organization of pyramidal cells in the hippocampus proper, as well as the predominantly unidirectional flow of information, produces a uniquely robust LFP signal that can be readily observed in animal models. This robust signal can be used to infer synchronous LFP activity with a relatively high degree of precision, as well as providing a reference point from which to investigate phase coding. As a result, a large body of work has focused on network synchrony and phase coding in relation to hippocampal LFPs, and the properties and mechanisms of these phenomena are relatively well characterized in comparison to other regions (Colgin, 2016; Drieu and Zugaro, 2019).

In humans, the hippocampus has predominantly been associated with episodic memory (Scoville and Milner, 1957; Vargha-Khadem et al., 1997), and recent evidence also suggests that prospective memory, such as the simulation of prospective episodes based on prior experience, is also hippocampus-dependent (Schacter et al., 2017). One defining characteristic of episodic memory is that it is anchored to a spatio-temporal context (Tulving, 1993). Thus, episodic memory typically includes details about where an event took place, and how the discrete components that comprise such events are ordered chronologically within the event space. Several aspects of hippocampal processing are ideally suited for the construction of episodic memory. For example, principal hippocampal cells, known as “place cells,” are known to encode information about the spatial location as an animal moves through physical space (O’Keefe and Dostrovsky, 1971), and spatial cognition has been linked to memory performance across a number of experimental paradigms in both animals and humans (Eichenbaum et al., 1999; Smith and Mizumori, 2006; Eichenbaum, 2017b). The hippocampus also plays an important role in temporal processing (Meck et al., 2013; Eichenbaum, 2014) including temporal pattern separation (Jacobs et al., 2013) and sequence generation (Buzsáki and Tingley, 2018). Importantly, both spatial and temporal sequencing mechanisms are known to require the synchronized coordination of oscillatory activity in the theta, gamma, and sharp-wave ripple bands (Buzsáki, 2006).

Schizophrenia has been associated with structural, neurochemical, and functional abnormalities of the hippocampal formation at all stages of disease progression (Heckers, 2001; Heckers and Konradi, 2002; Harrison, 2004). This includes decreases in synapse expression (Heckers, 2001; Harrison, 2004) and altered GABAergic signaling (Benes et al., 1998; Zhang and Reynolds, 2002) that are consistent with disturbed oscillatory activity. At the macroscopic level, episodic memory impairments have frequently been observed in individuals with schizophrenia (Rushe et al., 1999; Toulopoulou et al., 2003; Danion et al., 2005, 2007; Leavitt and Goldberg, 2009; Berna et al., 2016), and one study has also shown disturbed hippocampal activation in patients as they imagine future scenarios (D’Argembeau et al., 2008). These complex cognitive operations are difficult to measure in animals, but the more fundamental aspects that are thought to underlie episodic memory construction, such as place cells and sequential processing, can readily be investigated in preclinical models. Importantly, schizophrenia has also been associated with spatial memory impairments (Park and Holzman, 1992; Park et al., 1995; Glahn et al., 2003; Hanlon et al., 2006; Weniger and Irle, 2008; Fajnerová et al., 2014), and sequential processing deficits have also been observed in patients and first-degree relatives (Dickinson et al., 2007; Siegert et al., 2008; Nour et al., 2021).

The prefrontal cortex has been frequently implicated in schizophrenia pathophysiology (Selemon and Zecevic, 2015; Caballero et al., 2016), and it is known to have an important role in several cognitive processes that are disrupted in patients, such as working memory, executive control, and adaptive behavioral responses (Perlstein et al., 2001; Forbes et al., 2009; Eisenberg and Berman, 2010; Narayanan et al., 2013; Senkowski and Gallinat, 2015). In particular, dysfunction across the hippocampus-PFC pathway is correlated with a range of cognitive deficits in schizophrenia (Pantelis et al., 2003; Ziermans et al., 2012; Godsil et al., 2013; Cannon et al., 2015). Interactions between these regions are also thought to play a critical role in the consolidation of long-term episodic memory, spatial decision making, and the assimilation of new memories within pre-existing knowledge frameworks, or schema (Preston and Eichenbaum, 2013; Squire et al., 2015; Sigurdsson and Duvarci, 2016).

## Gamma Frequency Oscillations in The Hippocampus and Prefrontal Cortex

Disturbed gamma activity appears to be particularly pronounced in individuals with schizophrenia, and such disruptions have been observed during both cognitive task performance (Cho et al., 2006; Basar-Eroglu et al., 2007; Haenschel et al., 2009; Barr et al., 2010; Senkowski and Gallinat, 2015; Barr et al., 2017) and at rest (Andreou et al., 2015; Grent et al., 2018). Gamma frequency disturbances have also been observed in unmedicated, first episode patients and first-degree relatives, suggesting that it may be an endophenotype of the disorder (Uhlhaas and Singer, 2010; Williams and Boksa, 2010). Such disturbances have also been linked to a dysregulation of E/I balance in patients at several stages of illness progression (Grent et al., 2018).

The integrity of gamma activity has been associated with successful working memory performance, spatial cognition, selective attention, sensory gating, and the perceptual “binding” of discrete components into an integrated whole (Gray et al., 1989; Fell et al., 2003; Haenschel et al., 2009; Nyhus and Curran, 2010; Williams and Boksa, 2010; Nguyen et al., 2020). Current evidence also suggests that gamma activity is important for the temporal organization of information within local circuits (Von Stein and Sarnthein, 2000; Siegel et al., 2009; Moran and Hong, 2011), and for suppressing irrelevant circuit noise in control animals (Sohal et al., 2009). PV+ interneurons in particular have been identified as a critical component in this latter process (Sohal et al., 2009), consistent with proposals that widespread GABAergic disturbances in schizophrenia contribute to gamma-mediated working memory impairments (Lewis et al., 2005). Recent studies have also shown that dopamine modulation coordinates gamma activity in prefrontal regions (Lohani et al., 2019), again consistent with schizophrenia pathophysiology (Howes and Kapur, 2009).

In line with human studies, gamma disturbances have consistently been observed in a number of different animal models, including models of genetic risk (Fisahn et al., 2009; Deakin et al., 2012; Fejgin et al., 2014; Sauer et al., 2015; Zhao et al., 2021), neurodevelopmental models such as MIA (Dickerson et al., 2010, 2014; Nakamura et al., 2019; Schroeder et al., 2019; Lippmann et al., 2021) and MAM (Lodge et al., 2009) as well as a large number of NMDAR hypofunction models (Cunningham et al., 2006; Pinault, 2008; Dzirasa et al., 2009; Hakami et al., 2009; Lodge et al., 2009; Dickerson et al., 2010; Kittelberger et al., 2012; Caixeta et al., 2013). Taken together, such studies suggest that the integrity of gamma oscillations may be particularly sensitive to a diverse range of cellular and molecular disturbances, and may therefore represent a common physiological outcome of these disturbances at the network level. In general, the majority of these studies have shown evidence of increased gamma power at baseline, particularly among NMDAR hypofunction models (Bianciardi and Uhlhaas, 2021). This is consistent with studies showing excessive gamma activity in individuals with schizophrenia during working memory tasks (Barr et al., 2010).

In particular, within-animal studies of NMDAR blockade by either ketamine or MK-801 have provided more causal evidence that NMDAR disruptions alter cortical gamma activity. *In vivo* studies of acute NMDAR blockade have generally found a consistent pattern of results in hippocampal regions, with increased gamma power being reported as well as hyperactive behaviors as rats freely roamed around a familiar environment (Ma and Leung, 2000, 2007; Kittelberger et al., 2012; Caixeta et al., 2013; Ji et al., 2013; Nagy et al., 2016; Kealy et al., 2017; Lee et al., 2017; Sampaio et al., 2018). However, increases in hippocampal gamma power have been shown to occur independently of locomotor hyperactivity, indicating that elevated gamma power is not simply a reflection of hyperactivity (Lazarewicz et al., 2010; Caixeta et al., 2013). Furthermore, although administration of ketamine has also been shown to increase baseline, evoked, and induced gamma power in the hippocampus, the relative power of induced gamma, when compared to baseline recordings, was decreased (Lazarewicz et al., 2010). Similar increases in sound-evoked gamma oscillations were observed from LFP electrodes located in the CA1 region (Sullivan et al., 2015). Importantly, the same study obtained similar results from both surface EEG recordings and LFP probes, providing verification that in this case, non-invasive recording techniques reflected findings obtained from more invasive methods, a critical step in assessing the translatability of animal studies to humans (Sullivan et al., 2015).

Increases in cortical gamma power following acute NMDAR antagonism have also been observed in a number of *in vivo* studies (Pinault, 2008; Hakami et al., 2009; Kocsis, 2012; Kulikova et al., 2012; Phillips et al., 2012b; Jones et al., 2014; Molina et al., 2014; Lee et al., 2017; Hansen et al., 2019; Aguilar et al., 2021). In one study, however, the effects were dose-dependent, with the highest doses leading to decreased gamma power (Hiyoshi et al., 2014). Furthermore, although ongoing gamma was elevated in another study, both stimulus-evoked gamma and PPI were reduced, suggesting that sensory gating abnormalities associated with schizophrenia may be linked to a diminished ability to modulate gamma activity accordingly (Jones et al., 2014). Pre-treatment with antipsychotics has also been shown to reduce baseline gamma power in cortical regions, although only chronic pre-treatment attenuated increased gamma power following exposure to ketamine (Anderson et al., 2014), whereas acute doses had no effect (Jones et al., 2012). However, in a follow-up study, both ketamine and MK-801 administration resulted in a reduction of evoked gamma power in response to a pre-pulse stimulus. This effect was attenuated *via* administration of clozapine only, indicating that the distinct mechanisms of action associated with these antipsychotics have specific effects on either ongoing or evoked gamma activity (Hudson et al., 2016).

Studies conducted *in vitro* have also reported increases in induced gamma power in both hippocampal and prefrontal slices following systemic exposure to MK-801 (Kehrer et al., 2007; Lemercier et al., 2017), although there was no difference in spontaneous gamma activity (Lemercier et al., 2017). These effects were attenuated in a follow-up study *via* pre-treatment with the antipsychotic cariprarzine (Meier et al., 2020).

Other important factors to consider are the time course of drug action, the effects of downstream signaling cascades, and other compensatory or homeostatic processes that may not be captured by acute NMDAR blockade. For example, one study has reported that hippocampal gamma was unaffected following acute administration of MK-801 (Kalweit et al., 2017), in contrast to several studies showing elevated gamma activity (Ma and Leung, 2000, 2007; Kittelberger et al., 2012; Caixeta et al., 2013; Ji et al., 2013; Nagy et al., 2016; Kealy et al., 2017; Lee et al., 2017; Sampaio et al., 2018). However, in the Kalweit et al. (2017) study, *in vivo* recordings were taken either 1 or 4 weeks after exposure to the drug, suggesting that acute NMDAR hypofunction only has transient effects on gamma activity. Interestingly, this manipulation still resulted in both reduced LTP and theta/gamma cross-coupling at both time-points, indicating that acute NMDAR hypofunction may have more long–term effects on cross-frequency coupling. Studies of chronic exposure to NMDAR antagonists have reported a different pattern of results. For example, chronic administration of ketamine resulted in a steady decrease in hippocampal gamma power 2–4 weeks after treatment, and this coincided with decreased numbers of PV+ interneurons (Kittelberger et al., 2012). Paradoxically, however, animals with the greatest PV+ reductions had increased gamma power relative to animals with smaller PV+ reductions (Kittelberger et al., 2012). Reduced gamma power has been observed following chronic ketamine (but not MK-801) exposure in slices from the rodent prelimbic cortex, a region that is analogous to the human dorsolateral prefrontal cortex (McNally et al., 2013). Taken together, these studies indicate that chronic NMDAR hypofunction may result in a different pattern of gamma abnormalities when compared to more acute exposures, although more studies will be required to explore this possibility.

EEG and MEG studies of baseline gamma activity in patients with schizophrenia have reported mixed results, although acute administration of ketamine in healthy humans typically produces similar gamma increases to those observed in animal studies (for a systematic review see Bianciardi and Uhlhaas, 2021). It might therefore be expected that selective NMDAR knockout models may show a more similar pattern to schizophrenia patients, although surprisingly, such models have tended to show increased baseline gamma activity in hippocampal regions (Korotkova et al., 2010; Carlén et al., 2012; Tatard-Leitman et al., 2015), more in line with acute NMDAR blockade. These models did however manifest a range of cognitive and behavioral abnormalities that reflect schizophrenia symptoms, and auditory-evoked gamma was also reduced in the study by Tatard-Leitman et al. (2015). Induced gamma was also reduced in hippocampal slices from a mutant model lacking certain AMPA receptors subunits on PV+ interneurons, and this result appeared to proceed from imprecise spike timing (Fuchs et al., 2007).

MIA studies have shown that hippocampal gamma power at baseline was unaffected in both familiar and novel environments, but acoustic-evoked gamma and PPI were both reduced (Nakamura et al., 2019). Reduced gamma power has also been observed in an MIA model during decision making and memory tasks, although this reduction was only observed in female offspring (Schroeder et al., 2019). Reduced gamma coherence between the PFC and hippocampus has also been associated with diminished PPI, although gamma power was unaffected (Dickerson et al., 2010, 2014). The temporal spiking of neurons in relation to gamma oscillations was also disturbed in the MIA model (Dickerson et al., 2010). Similar reductions of gamma coherence were observed in MIA animals prior to repetitive transcranial magnetic stimulation (rTMS), although this effect was partially attenuated following the rTMS protocol, suggesting that this may be a viable treatment option (Lippmann et al., 2021). Taken together, these studies suggest that MIA leads to reductions in either gamma power or coherence during specific tasks, and these disruptions may have important functional implications, for sensory gating in particular.

In another neurodevelopmental model, exposure to MAM on GD 17 has also been shown to decrease stimulus-evoked gamma power in offspring during a latent inhibition paradigm, and this was correlated with decreased numbers of PV+ interneurons in hippocampal and prefrontal regions (Lodge et al., 2009).

Models of genetic risk have also shown abnormal gamma activity. Gamma power during active exploration was increased in a *Df(h15q13)/+* model, although relative evoked gamma power in response to auditory stimulation was reduced (Fejgin et al., 2014), a pattern that reflects aberrant gamma activity frequently observed in schizophrenia patients (Light et al., 2006; Spencer et al., 2008; Brenner et al., 2009). Reductions of gamma power have also been observed in hippocampal slices from a dysbindin-1 model (Zhao et al., 2021). However, in another *in vitro* study, hippocampal gamma was indistinguishable from controls in a model of LPA-1 deficiency, although gamma power in superficial layers of the entorhinal cortex was significantly increased (Cunningham et al., 2006). There are a number of potential explanations for these different results, but the most likely is that the regulation of gamma activity in hippocampal regions may be affected by network activity that originates outside the hippocampus proper and that these more complex mechanisms are not captured in isolated slices (Cunningham et al., 2006). In support of this proposal, emerging evidence that entorhinal cortex-hippocampus pathways are critical for the organization of information transfer at gamma frequencies suggests that the integrity of EC transmission is likely to exert important effects on hippocampal gamma power and synchrony (Fernández-Ruiz et al., 2017, 2021).

Models targeting neuregulin signaling have also shown a range of induced gamma abnormalities, including reduced gamma frequency (Deakin et al., 2012) and power (Fisahn et al., 2009) in hippocampal slices. Neuregulin signaling has been shown to be important for the synchronization of network activity in the prefrontal cortex *in vivo* (Hou et al., 2014; Barz et al., 2016), and increases of induced gamma power that occur in wildtype animals were absent in mutant mice lacking ErbB4 receptors on interneurons located in frontal regions (Hou et al., 2014). Stimulus-evoked gamma is also reduced in mice with the Neurogulin-1 genetic susceptibility (Barz et al., 2016). DISC-1 models have shown disturbed synchrony in the gamma range that was associated with disrupted PV+ interneurons (Sauer et al., 2015), and recent dual-hit models (DISC1 and MIA) have also shown disorganized temporal spiking in relation to oscillatory activity in the gamma range (Hartung et al., 2016; Chini et al., 2020).

Several studies using animal models have also demonstrated that the familiarity of the task or recording environment is likely to exert important effects on gamma activity, suggesting that gamma frequency oscillations may play an important role in the reallocation of attentional resources in response to novelty. For example, a reduced shift in the preferred gamma firing phase of single cells located in the CA1 region in response to novelty has been observed in a DISC-1 model of genetic risk, and principal cells were more strongly phase- locked to both gamma and theta oscillations, specifically in novel environments (Kaefer et al., 2019). Novelty-induced irregularities were also observed in a genetic model of NMDA hypofunction (SRKO), in which the power of background gamma oscillations in frontal regions was increased prior to a social recognition task. When another animal was introduced to the testing arena, however, there was an attenuated increase in gamma power relative to controls, associated with reduced social recognition (Aguilar et al., 2021). These disruptions may be due to neuregulin-induced increases in dopamine signaling, as D4 dopamine receptor agonists increased gamma activity in hippocampal slices, and both NRG-1 and D4 receptor types are co-expressed on PV+ interneurons (Andersson et al., 2012). In another study that compared hippocampal-PFC gamma synchrony between wildtype and hyperdopaminergic (DAT-KO) mice in both novel and familiar environments, gamma synchrony between the hippocampus and PFC was initially high in both groups in the home environment. This was attenuated in the control group when animals subsequently explored a novel environment, resulting in elevated inter-regional gamma synchrony in the mutant group when compared to controls (Dzirasa et al., 2009). Although these studies are inconsistent in regards to the enhancement or attenuation of gamma activity in response to novelty, they all suggest that abnormal gamma activity during rest is likely to be an important factor when interpreting such results. Further support for this idea has been provided by studies demonstrating elevated CA1 gamma activity in a ketamine model when animals are well habituated to the environment (Caixeta et al., 2013). Increased hippocampal gamma activity reminiscent of REM sleep has also been observed in a DAT-KO model as animals explored a novel environment, an effect that was normalized *via* treatment with the antipsychotic haloperidol (Dzirasa et al., 2006). Taken together, these studies suggest that schizophrenia may be associated with inappropriate state-dependent gamma processing, which may disrupt the facilitation of long term potentiation (LTP) in response to novelty when learning is likely to be most beneficial (Li et al., 2003).

Overall, the evidence from animal models is largely consistent with human studies showing that gamma activity is disturbed in individuals with schizophrenia. The majority of studies have shown evidence of increased baseline gamma, whereas stimulus-evoked and induced gamma were more frequently, but not always, reduced. This suggests that abnormal gamma activity in response to changing environmental and task demands may underlie at least some of the sensory gating and task switching disturbances that have been associated with the disorder.

## Sharp Wave Ripples and Replay

Sharp wave ripples (SPW-Rs) involve an irregular pattern of large amplitude waves that are typically present in hippocampal regions during slow-wave sleep, or when animals are awake but immobile (Buzsáki, 1986, 2015). These sharp wave events typically last for around 40–100 ms and are accompanied by a “ripple” oscillation that occurs above the gamma frequency range, between 100 and 200 Hz. The SPW-R is the LFP event that co-occurs with a neuron-level phenomenon known as a replay, whereby sequences of place field activity that has previously occurred during active exploration are reactivated (Pavlides and Winson, 1989; Wilson and McNaughton, 1994; Lee and Wilson, 2002). The reactivation of sequential spiking activity that occurs during SPW-Rs occurs in a time-compressed manner such that the representation of events occurs in a timeframe that is suitable for the induction of synaptic plasticity (Davidson et al., 2009). These reactivation patterns are most prominent during the first few hours after learning, and they are thought to contribute to the consolidation of newly acquired information and the subsequent transfer of memory from the hippocampus to more permanent storage in neocortical regions. Consistent with this proposal, perturbation of SPW-R activity during post-learning sleep in rodents has been shown to impair performance on spatial memory tasks (Girardeau et al., 2009; Ego-Stengel and Wilson, 2010). Similarly, stimulation of reward regions in response to SPW-R related place cell activity during sleep has been shown to induce an artificial place/reward association, providing compelling evidence that replay during sleep is functionally important for goal-related spatial memory (De Lavilléon et al., 2015). Replay events have also been shown to predict future trajectories (preplay) and so they may also have a role in planning (Pfeiffer and Foster, 2013).

Disordered ripple events have been observed in both a methylazoxymethanol acetate (MAM) neurodevelopmental model (Phillips et al., 2012a), and a DISC-1 genetic model (Altimus et al., 2015). Other studies, using a genetically modified calcineurin animal model which has been shown to reproduce several phenotypes associated with schizophrenia (Miyakawa et al., 2003), have also demonstrated a substantial increase in hippocampal SPW-R events in mutant animals during awake rest, as well the elimination of sequential replay (Suh et al., 2013). Furthermore, in a recent *in vitro* study, the temporal structure of SPW-R events was shown to be altered in hippocampal slices obtained from MIA animals (Gao et al., 2019). These findings are all consistent with the hypothesis that pathological ripple activity could be involved in schizophrenia (Buzsáki, 2015). Recent advances have also made it possible to investigate SPW-R events in humans (Liu et al., 2019), and early evidence from schizophrenia patients indicates that replay is diminished, although ripple activity is enhanced relative to control subjects. in schizophrenia patients (Nour et al., 2021). These findings are consistent with the animal literature, although further work will be required to determine how these changes affect processes such as memory consolidation and planning. Furthermore, it is not clear what underlying changes produce the alterations in SPW-R events that are described here. For example, do they reflect subtle changes in circuitry or functional connectivity, or are they simply a response to a general loss of inhibition?

## Theta Frequency Oscillations in The Hippocampus and PFC

Although early studies of disturbed oscillatory activity in individuals with schizophrenia have focused predominantly on higher-frequency oscillations (Uhlhaas and Singer, 2010), more recent work has demonstrated that disturbances in the lower-frequency theta band are also common (Schmiedt et al., 2005; Siekmeier and Stufflebeam, 2010; Kirihara et al., 2012; Frantseva et al., 2014; Griesmayr et al., 2014; Andreou et al., 2015; Cousijn et al., 2015; Di Lorenzo et al., 2015; Garakh et al., 2015; Kim et al., 2015; Javitt et al., 2018; Ryman et al., 2018; Adams et al., 2020). Theta oscillations are thought to coordinate long-range communication across regions (Von Stein and Sarnthein, 2000; Moran and Hong, 2011) and theta frequency disturbances are therefore likely to be critical for a wide range of complex cognitive processes that require the integration of both higher and lower order processes across distributed networks. Theta oscillations in hippocampal and prefrontal regions have been extensively studied in both humans and non-clinical animal models, and theta activity in these regions has been associated with an exceptionally diverse range of cognitive operations, including episodic, spatial, and working forms of memory, sequential processing, adaptive learning, error monitoring, relational binding, social cognition, and flexible decision making. These studies have been comprehensively reviewed elsewhere (Hasselmo, 2005; Nyhus and Curran, 2010; Buzsáki and Moser, 2013; Colgin, 2013, 2016; Cavanagh and Frank, 2014; Hasselmo and Stern, 2014; Buzsáki and Tingley, 2018; Herweg et al., 2020; Karakaş, 2020).

The biophysical mechanisms underlying theta oscillations have also been extensively studied in non-clinical animal models, and such studies have provided a framework from which to understand the likely role of schizophrenia pathophysiology in disturbed oscillatory activity (Lisman and Buzsáki, 2008). For example, the generation and maintenance of the hippocampal theta rhythm involve several neurotransmitter systems that are known to be disturbed in schizophrenia, including the glutamate, GABA, dopamine, and acetylcholine systems (Freund and Antal, 1988; Stewart and Fox, 1990; Howes and Kapur, 2009; Losonczy et al., 2010; Moghaddam and Javitt, 2012; Nakazawa et al., 2012; Gonzalez-Burgos et al., 2015; Drieu and Zugaro, 2019; Caton et al., 2020). Furthermore, the regulation of local inhibitory networks has also been shown to exert profound effects on theta synchrony (Cobb et al., 1995; Kamondi et al., 1998; Goutagny et al., 2009). In particular, PV+ interneurons that target the peri-somatic regions of principal cells appear to play an important role in the temporal coordination of rhythmic LFPs within the theta range, as well as the temporal spiking profile of single cells relative to distinct theta phases of the theta cycle (Wulff et al., 2009; Stark et al., 2013; Amilhon et al., 2015). Findings from animal models of schizophrenia risk are generally consistent with these findings, indicating that theta disturbances frequently co-occur with disturbed GABAergic signaling, particularly at the site of PV+ interneurons (Lodge et al., 2009; Korotkova et al., 2010; Ducharme et al., 2012; Del Pino et al., 2013; Dickerson et al., 2014; Sauer et al., 2015; Nakamura et al., 2019).

To date, a broad range of abnormalities in theta activity in hippocampal and prefrontal regions have been described in animal models of schizophrenia, with evidence of both enhanced and reduced theta power, coherence, and synchrony. Models of NMDAR hypofunction, including both acute exposure and selective knockout models, have shown evidence of decreased baseline theta power in hippocampal regions (Korotkova et al., 2010; Lazarewicz et al., 2010; Kalweit et al., 2017). Event-related theta power in the hippocampus was also significantly reduced following sub-chronic exposure to ketamine when animals were tested 6 months after cessation of the drug exposure protocol, suggesting that chronic NMDAR hypofunction over a discrete time period can exert more permanent effects on circuitry (Featherstone et al., 2012). Acute administration of ketamine, however, led to layer-specific modulation of theta power in CA1 as animals freely moved around the recording apparatus (Caixeta et al., 2013). These latter data are consistent with evidence that theta properties vary systematically according to the precise location of recording electrodes in the hippocampus (Buzsáki et al., 1985; Brankačk et al., 1993; Lubenov and Siapas, 2009), and suggest that quite small changes in experimental procedures could influence the results. Increased theta power has also been observed in a genetic model of the disorder that knocks out a neuregulin receptor (ERBb4), a critical receptor for the integrity of fast-spiking interneurons. This increase in theta power co-occurred with increased intra-regional coherence across the hippocampal circuit but decreased theta synchrony between the hippocampus and PFC (Del Pino et al., 2013).

Disrupted phase-locking of single cells located in either the PFC or the hippocampus to the hippocampal theta rhythm has also been observed in both a DISC1 and a 22q11 deletion *(Df(16)^A + /-^)* model, including decreases in both the phase-locking strength of individual cells, as well as the synchronization of preferred locking phase at the network level (Sigurdsson et al., 2010; Kaefer et al., 2019). In the *Df(16)^A + /-^* model, these disturbances were also associated with reduced LFP coherence between the hippocampus and the PFC, as well as working memory impairments (Sigurdsson et al., 2010). Similar reductions were observed in an alternative model targeting the 22q11.2 deletion, in which the deficiencies at the site of the ZDHHC8 gene resulted in reduced axonal growth during early development (Mukai et al., 2015).

Prelimbic theta synchrony has also been shown to be reduced in a DISC-1 model, although this effect appeared to be driven by reduced theta power in the hippocampus, although coherence was unaffected (Sauer et al., 2015). Disorganized hippocampal theta oscillations and reduced hippocampal/PFC theta synchrony have recently been observed in neonates exposed to a dual-hit procedure (DISC-1 and MIA). However, theta synchrony was subsequently augmented in pre-juveniles, suggesting that theta activity is likely to be sensitive to ongoing developmental processes (Hartung et al., 2016). Furthermore, unlike the single-hit genetic model (DISC1), MIA did not affect synchrony on its own, suggesting that the time-course of disruptions associated with each model is different and that such disruptions interact with each other in a complex fashion (Hartung et al., 2016). Interestingly, MIA has also been shown to delay the maturation of GABAergic transmission from predominantly depolarizing to hyperpolarizing (Corradini et al., 2018; Fernandez et al., 2018), which suggests that the precise time-course of such developmental shifts could potentially play a crucial role in the emergence of coordinated network synchrony later in life.

Single hit MIA models have generally shown a number of theta frequency disturbances once offspring reach maturity, including increased theta power at baseline, but reductions in evoked theta power (Nakamura et al., 2019). Increased theta power has been observed to occur in conjunction with diminished synaptic inhibition in hippocampal slices following an MIA manipulation (Ducharme et al., 2012). Decreased coupling between hippocampal and prefrontal regions has also been observed in an anesthetized MIA model, although coherence was similar to controls (Lippmann et al., 2021). However, theta coherence and the phase locking of PFC cells to hippocampal theta have been shown to be disturbed in an MIA model during waking behaviors (Dickerson et al., 2010, 2014), similar to findings reported in genetic-risk models (Sigurdsson et al., 2010). Furthermore, abnormal theta synchrony between these regions was attenuated in the MIA model following administration of the antipsychotic clozapine, although local increases in theta power were only observed in the PFC, suggesting that long-range coherence was more likely to be mediated by increased PFC theta synchrony than local changes in the hippocampus (Dickerson et al., 2012).

Reductions in PFC theta activity have also been reported in the MAM model of schizophrenia during a fear conditioning paradigm, while theta activity in the hippocampus was unchanged, again suggesting that theta disruptions in the PFC may be driving the functional dysconnectivity between these regions (Lodge et al., 2009). The same MAM model has previously been shown to produce both hippocampal hyperactivity and a subsequent hyperdopaminergic state that could be attenuated *via* inactivation of the ventral hippocampus, suggesting that hippocampal signaling may exert important effects on theta activity in downstream regions *via* dopamine modulation (Lodge and Grace, 2007, 2008). This is consistent with proposals that GABAergic disturbances in hippocampal regions are likely to have important effects on downstream dopamine signaling (Sonnenschein et al., 2020). However, theta phase synchrony between the hippocampus and PFC was not disrupted in a hyperdopaminergic model of the disorder created by knocking out a key dopamine transporter gene, suggesting that dopamine irregularities are not likely to be the primary mechanism of dysfunctional theta activity between these regions (Dzirasa et al., 2009).

Interestingly, infusion of dopamine into the PFC of naïve, anesthetized rats initiated similar increases in theta phase coherence and synchrony between the PFC and hippocampus to those observed during successful rule learning (Benchenane et al., 2010). This suggests that dopamine signaling in response to salient stimuli and prediction error may play a critical role in coordinating phase synchrony between the hippocampus and PFC and that such synchrony supports adaptive learning. Further support for this hypothesis has been provided by both human and rodent studies showing that lower frequency oscillations (<12 Hz) are important for adaptive behavioral adjustments in response to error detection (Narayanan et al., 2013). Hyperdopaminergic activity in schizophrenia may, therefore, contribute to inefficient cognitive task switching in response to current environmental and motivational demands.

In support of this hypothesis, a reduced novelty-induced shift in the preferred theta phase of CA1 cells has been observed in a DISC-1 model, accompanied by disturbed theta coordination at the network level during exploration (Kaefer et al., 2019). The DISC-1 model has also been associated with a number of dopamine signaling abnormalities (Trossbach et al., 2016). It has been proposed that hippocampal-PFC theta coherence may reflect sustained attention rather than working memory, as impaired spatial working memory performance could be predicted by either low gamma or beta coherence in a genetic risk model (gria1^−/−^), while theta coherence was only disturbed in a novelty recognition paradigm (Bygrave et al., 2019). Given that several studies have also documented abnormal theta activity during resting states in both patients with schizophrenia and animal models of the disorder (Karbasforoushan and Woodward, 2012; Del Pino et al., 2013; Kaefer et al., 2019), these findings indicate that the dynamic modulation of theta activity in response to salient changes in either contextual cues or task demands may be a more critical component of schizophrenia pathology than simple hypo- or hypersynchrony within and between these regions.

In human studies, reduced theta power and diminished theta phase coupling between the mPFC and the medial temporal lobe have been observed in individuals with schizophrenia, and this was correlated with both memory performance and abnormal GABA_A_ receptor expression in the schizophrenia group (Adams et al., 2020). Both the coherence of theta oscillations between hippocampal and prefrontal regions and the synchronous phase locking of PFC neurons to the hippocampal theta rhythm have been associated with spatial and working memory performance (Zielinski et al., 2019) as well as successful rule learning (Benchenane et al., 2010) in non-clinical rodent models. Tests of animal models of schizophrenia that have included a cognitive task have generally been consistent with these findings, demonstrating that reduced theta coupling between the hippocampus and PFC is correlated with both spatial working memory deficits (Dzirasa et al., 2009; Sigurdsson et al., 2010; Del Pino et al., 2013) and reduced pre-pulse inhibition (Dickerson et al., 2010). Reductions in hippocampal theta power (Korotkova et al., 2010) and frequency (Fejgin et al., 2014) have also been associated with spatial memory deficits in animal models. In one model, however, impaired recognition memory was correlated with enhanced hippocampal/PFC coupling, although in that study, LFP activity was recorded while animals were under-anesthesia, which is unlikely to reflect theta activity that is directly associated with the cognitive task (Hartung et al., 2016). Additional studies will be required to clarify some of these outstanding issues, although in general, these studies suggest that targeting theta activity in hippocampal and prefrontal regions may be a promising avenue for future research into cognitive disorganization in schizophrenia.

Intriguingly, another study using a neurodevelopmental model of schizophrenia has demonstrated that targeted cognitive training during adolescence can normalize theta synchrony within hippocampal regions and that this normalization coincided with a rescue of the cognitive deficits that typically emerge post-adolescence (Lee et al., 2012). This suggests that targeting basic level mechanisms that support learning and memory during critical developmental periods is a viable strategy for preventing the development of schizophrenia in high-risk individuals, although it remains unclear whether directly enhancing theta coupling between hippocampal and prefrontal regions can also prevent pathological trajectories. In this vein, however, a recent study has shown that non-invasive electrical stimulation to frontal regions can promote theta synchrony in schizophrenia patients and that this effect was accompanied by improved cognitive control (Reinhart et al., 2015).

Overall, the evidence from animal models supports the proposal that disturbed theta activity may be related to several cognitive deficits observed in the disorder, although the pattern is complex. While NMDAR hypofunction models have generally shown evidence of reduced theta power, models of both genetic and environmental insults during early neurodevelopment have produced more variable results. This aside, coordinated synchrony between the hippocampus and PFC and abnormal phase-locking of single cells to the theta rhythm has been consistently observed across several studies suggesting that these processes may be a viable target for novel interventions.

## Theta/Gamma Cross-Coupling

LFP oscillations at gamma frequencies are often nested within the slower theta rhythm during specific behaviors, with higher gamma amplitudes typically coupled to the peak of the theta oscillation (Csicsvari et al., 2003; Belluscio et al., 2012; Colgin, 2016). This phenomenon, known as cross-frequency coupling, is thought to play an important role in the temporal organization of information during working and episodic memory processes (Lisman and Idiart, 1995; Lisman, 2005; Lisman and Buzsáki, 2008; Lisman and Jensen, 2013). Support for this hypothesis has been obtained in a number of studies showing that the strength of theta/gamma cross-coupling is increased during successful memory performance in rodents (Tort et al., 2009; Shirvalkar et al., 2010), monkeys (Jutras et al., 2009), and humans (Sederberg et al., 2006; Axmacher et al., 2010; Maris et al., 2011; Heusser et al., 2016).

It has also been proposed that the theta/gamma neural code may function as a neural syntax, with each gamma oscillation representing a single “word,” while the theta oscillation works to organize the sequential order of such “words” into meaningful sentences (Lisman and Buzsáki, 2008; Buzsáki, 2010). Disturbed cross-frequency coupling has thus been linked to cognitive disorganization in schizophrenia (Lisman and Buzsáki, 2008), although experimental evidence for this proposal has been challenging to obtain. For example, no differences in cross-frequency coupling were observed when patients performed a simple auditory processing task (Kirihara et al., 2012), and although global theta/gamma cross-coupling was diminished in another study, it was actually enhanced for patients across electrodes located specifically in frontal temporal regions (Allen et al., 2011). More recently however, impaired theta/gamma cross-coupling in the PFC has been observed in patients while they performed a working memory task, and this was associated with poor task performance when compared to control subjects (Barr et al., 2017). Interestingly, peak gamma power for individual items within a sequence has also been shown to be organized sequentially according to distinct theta phases in healthy humans (Heusser et al., 2016), suggesting that disturbed phase coupling could be involved in the disorganization of temporal sequencing.

Hippocampal theta/gamma coupling has been investigated in several animal models of the disorder. In general, coupling deficits have been reported with the hippocampus itself (Caixeta et al., 2013; Kalweit et al., 2017). Administration of ketamine has been shown to alter hippocampal theta/gamma cross-coupling in a dose-dependant manner, with increased coupling evident for the lowest dose (25 mg/kg), but diminished coupling at the highest dose (75 mg/kg; Caixeta et al., 2013). In another study that used an alternative NMDA antagonist model (MK801), hippocampal theta/gamma cross-coupling was transiently disrupted during a high-frequency stimulation protocol designed to induce LTP, and this uncoupling co-occurred with diminished theta power, whereas gamma activity remained uninterrupted (Kalweit et al., 2017). Previous studies have demonstrated that theta/gamma coupling is highly correlated with LTP induction (Bikbaev and Manahan-Vaughan, 2007, 2008), and given that hippocampal LTP was also profoundly diminished following the transient NMDA blockade, it is possible that disrupted cross-frequency coupling reflects aberrant plasticity processes (Kalweit et al., 2017). However, it remains unclear whether disturbed coupling is a cause or effect of impaired synaptic plasticity, or whether reduced coupling in these models is associated with cognitive deficits.

Diminished theta/gamma phase coupling within both the hippocampus and prefrontal cortex has also been observed in an NMDA hypofunction model (NR1 KD) as animals explored a novel environment, although inter-regional phase coupling was enhanced, suggesting that hyper-coupling between these regions could also be involved in pathological outcomes (Dzirasa et al., 2009). Enhanced cross-coupling between these regions was also observed in a dual-hit model (DISC-1 and MIA) under anesthesia, and although no differences were observed in either single-hit models in that study (Hartung et al., 2016), enhanced coupling was observed in another single-hit MIA model (Lippmann et al., 2021). This enhanced coupling furthermore was attenuated when animals were pre-treated with an rTMS protocol (Lippmann et al., 2021). The enhanced coupling has also been observed in naïve animals following stimulation of dopamine cells in the VTA (Lohani et al., 2019), suggesting that hyperdopaminergic activity in schizophrenia may also play a role.

Although the gamma rhythm has historically been conceptualized as a singular rhythm that encompasses a broad frequency range, recent reports suggest that gamma frequencies may be better conceptualized as two distinct frequency bands, with low gamma activity occurring at frequencies between 30 and 60 Hz, whereas high gamma occurs between 60 and 100 Hz (Colgin et al., 2009). These distinct bands are thought to have complementary functions in the hippocampus and may allow for the integrated organization of internally and externally generated information arriving from different sources. Thus, high gamma activity frequently occurs around the peak of the theta oscillation, and is thought to play an important role in the encoding of sensory information arriving from the EC, while low gamma tends to occur during the descending phases of the theta oscillation, and has been predominantly associated with memory retrieval processes originating in CA3 (Colgin et al., 2009; Schomburg et al., 2014). CA1 low gamma also predominantly co-occurs with sequential processing that sweeps ahead of the animal’s current location, suggesting that it is preferentially involved in the prospective coding of future locations, whereas high gamma appears to represent the animal’s current location in real time (Senior et al., 2008; Zheng et al., 2016), as well as during retrospective encoding of recently visited locations (Bieri et al., 2014). Although it currently remains unclear whether low and high gamma typically co-occur during a single theta cycle, or whether separate theta cycles preferentially represent either future and present locations depending on the animal’s current situation and goals (Colgin et al., 2009; Zheng et al., 2016), these findings suggest that cross-coupling may be important for the organized integration of new information within existing schemas. This coding scheme could also have important implications for aberrant source monitoring in schizophrenia (Brébion et al., 2000; Martin et al., 2014), potentially shifting the emphasis from externally generated sensory information to internally generated representations, and *vice versa*. At the present time, however, it is unclear how the high/low gamma relationship is influenced by schizophrenia or is affected in animal models of the disorder.

## Hippocampal Phase Precession and Theta Sequences

The theta rhythm is not only an indicator of synchronous neural activity, but it also serves as a reference signal against which temporal, or phase coding of information can occur. Theta phase precession is a form of temporal coding that was first observed in CA1 place cells as animals moved along a linear track. In addition to spatial rate coding, which produces localized “place fields”, it was noticed that the firing phase of these cells, referenced to the underlying theta-frequency LFP oscillation, changed systematically from a later to earlier phases of successive theta cycles as an animal advanced across a place field (O’Keefe and Recce, 1993; Skaggs et al., 1996). As a result, the firing phase of a cell provides information about where the animal is located within a place field, over and above that of the conventional rate code, and several studies have confirmed that this phase code is a more robust predictor of an animal’s current location than the rate code alone (Jensen and Lisman, 2000; Huxter et al., 2003; Tingley and Buzsáki, 2018).

While phase precession describes location-dependent changes in the spiking activity of single cells, it also has important implications for sequential processing at the network level. When several cells with overlapping place fields are co-active, the phase precession of individual cells produces an emergent phenomenon known as a “theta sequence” (Foster and Wilson, 2007), wherein recently experienced event sequences occurring at behavioral timescales are preserved and compressed within a single theta cycle (~120 ms), a timescale that is suitable for the induction of synaptic plasticity (Skaggs et al., 1996; Bi and Poo, 1998; Dan and Poo, 2004). Theta sequences have thus garnered considerable interest as a mechanism of sequential memory encoding and storage (Skaggs et al., 1996; Dragoi and Buzsáki, 2006; Jaramillo and Kempter, 2017; Buzsáki and Tingley, 2018; Drieu and Zugaro, 2019). Several studies have now confirmed that theta sequences rapidly emerge during active exploration of an environment, although additional network synchronization is required to ensure that critical phase precession properties, such as the starting phase and slope of precession, are relatively coherent across co-active cells (Foster and Wilson, 2007; Schmidt et al., 2009; Feng et al., 2015).

Both phase precession and theta sequences have now been observed in a range of experimental conditions, including tasks that require goal-planning and decision-making (Johnson and Redish, 2007; Gupta et al., 2012; Wikenheiser and Redish, 2015), as well as several paradigms that don’t include a spatial component (Lenck-Santini et al., 2008; Pastalkova et al., 2008; Royer et al., 2012; Cei et al., 2014). Importantly, hippocampal phase coding has also been associated with the sequential integration of sound and odor cues (Terada et al., 2017), as well as internally generated states (Takahashi et al., 2014; Wang et al., 2015). These findings suggest that theta sequences are involved in the complex construction of mental maps, an important component of both episodic memory and decision making (Kaplan et al., 2017). Interestingly, the developmental emergence of theta sequences has recently been shown to coincide with the maturation of hippocampal memory in rodents (Muessig et al., 2019), providing compelling evidence that theta sequences may serve as a neural substrate for episodic memory traces more generally. Recent studies have also demonstrated that theta sequences are associated with episodic memory and sequential planning in humans (Heusser et al., 2016; Kaplan et al., 2020), and direct evidence of phase precession has also been confirmed in single cell recordings from human subjects performing a virtual reality navigation task (Qasim et al., 2020).

**Figure 1:**
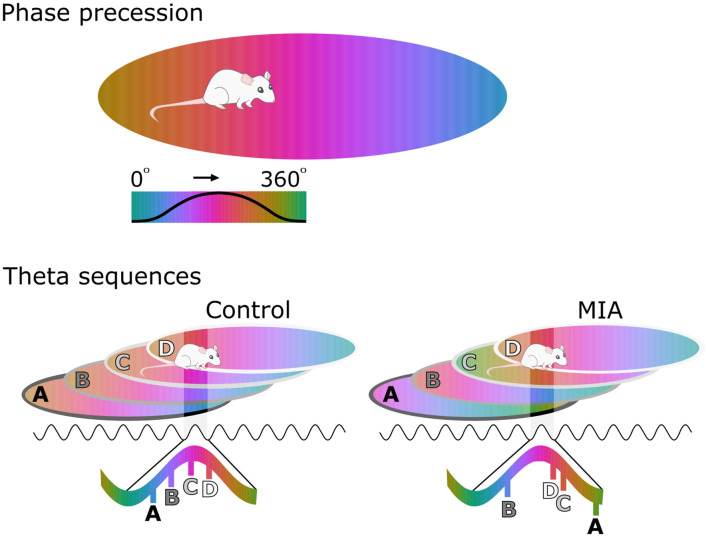
Disorganized phase coding of hippocampal place cells produces disordered theta sequences in maternal immune activation (MIA) animals. The upper cartoon illustrates phase coding occurring as an animal crosses a place field, with phase color-coded. As the animal enters the place field, the cell spikes at late phases of the theta cycle, but spiking processes towards earlier phases as the animal traverses the field. The lower cartoon demonstrates how theta sequences emerge as a result of phase precession in several cells with overlapping place fields. In the control example, the starting phase of precession is coordinated at the network level, resulting in ordered theta sequences that are concentrated along a portion of a theta cycle. Here cell A fires first during the theta cycle because the animal is exiting this place field. In contrast Cell D fires last, because the animal is entering this field. In the MIA example, starting phase varies from cell to cell, resulting in disordered sequences that are also spread further across the theta cycle.

Hippocampal phase precession has only recently been investigated in a model of schizophrenia risk. In this study, the firing of individual pyramidal cells in the CA1 region of MIA animals displayed what appeared to be normal phase precession as these animals moved through that cell’s place field. On closer examination, however, the starting phase of this precession as an animal enters a new place field was considerably more variable between-cells in MIA animals than in controls (Speers et al., 2021). An important theoretical consequence of this variability is that the sequence of place fields (or other experiences) that an animal encounters would be replayed in a disordered manner during each theta sequence (Figure 1). To test this hypothesis, the correlations between the spike time difference of simultaneously recorded cell pairs and the distance between their respective place fields were determined. Results showed that there was a significant positive correlation between these two measures in the control cells, as would be expected if theta sequences are functioning normally. In contrast, there was no such relationship in the MIA cells indicating that theta sequences were disordered in the MIA group (Speers et al., 2021). To illustrate the effect of this change, in MIA animals a sequence experienced in the order ABCD would be encoded and recalled in a disordered fashion, for example as BDCA.

In addition to disordered theta sequences, increased starting phase variability should result in reduced clustering of sequential spiking within each consecutive theta cycle, provided that individual cells do not precess a full 360 degrees (Schmidt et al., 2009). This could potentially allow spikes from one cycle to become erroneously associated with those in the next cycle, further corrupting the sequential order of experience, as well as distorting the segmentation of experience into discrete events (Gupta et al., 2012). An analogy for this phenomenon is that the pause in firing that normally occurs between cycles serves as “punctuation” by separating out units of meaningful information. This lack of “punctuation,” if it occurs in schizophrenia, may contribute to a disintegration of event boundaries (Lisman and Buzsáki, 2008; Richmond et al., 2017), consistent with evidence that event segmentation is disrupted at both lower and higher order levels among individuals with schizophrenia (Zalla et al., 2004; Coffman et al., 2016).

Two previous studies have also provided indirect evidence that the phase coding may be disrupted in other animal models of schizophrenia, although phase precession itself was not explicitly investigated. In one study, the phase-locking preference of CA1 cells to theta was more variable in a DISC-1 model (Kaefer et al., 2019), which would be a logical consequence of a more variable starting phase. Another study has demonstrated that administration of PCP, which has been shown to induce transient schizophrenia-like symptoms in healthy individuals and to exacerbate symptoms in patients, disrupts the precise spike timing of place cell pairs relative to the theta rhythm without disrupting other place field properties (Kao et al., 2017). Both of these studies are consistent with the findings outlined in Speers et al. (2021), suggesting that disorganized phase coding mechanisms potentially occur in other models of schizophrenia. Furthermore, although the precise mechanisms of phase precession and theta sequences remain to be elucidated, several animal models of schizophrenia have shown evidence of basic-level disturbances that are consistent with a discoordination of phasic spiking, with current evidence pointing towards PV+ interneurons as a critical factor (Lodge et al., 2009; Ducharme et al., 2012; Royer et al., 2012; Del Pino et al., 2013; Dickerson et al., 2014; Drieu and Zugaro, 2019).

Phase precession has been shown to occur in regions outside of the hippocampus, suggesting that phase coding mechanisms could be important across a wider distributed network. For example, phase precession has been documented in the prefrontal cortex (Jones and Wilson, 2005), as well as in subcortical areas that are likely to be important for dopamine regulation, such as the lateral septum, the striatum, and the ventral tegmental area (Lansink et al., 2009; Luo et al., 2011; van der Meer and Redish, 2011; Tingley and Buzsáki, 2018). In turn, striatal dopaminergic concentrations have been shown to be strongly influenced by the synchronization of GABAergic micro-circuits in a computational model, suggesting that dopamine might have a wider modulatory role in the coordination of phasic spiking at the network level (Humphries et al., 2009).

Finally, if theta sequences provide the biophysical scaffolding that supports the encoding and storage of temporally extended memories, then a disruption of this system could have profound implications for learning and memory processes, as well as the disorganization of thought that occurs in the disorder (Lisman and Buzsáki, 2008). Sequential processing deficits have frequently been documented in schizophrenia patients, their first-degree relatives, and other at-risk individuals, including disturbances of temporal order judgment and impaired sequence learning (Dickinson et al., 2007; Lisman and Buzsáki, 2008; Pedersen et al., 2008; Siegert et al., 2008; Meck et al., 2013; Ciullo et al., 2016; Eichenbaum, 2017a; Thoenes and Oberfeld, 2017). Such deficits also appear to be independent of other cognitive impairments (Ciullo et al., 2016), suggesting that they may be a primary feature of the disorder and a potential trait marker (Andreasen et al., 1999). A fundamental disorganization of sequential processing mechanisms could furthermore affect a wide range of cognitive processes that have been shown to be disturbed in schizophrenia (Barch and Ceaser, 2012; Thoenes and Oberfeld, 2017), and which can be effectively modeled in animals. Additional studies will be required to establish a more direct link between disrupted phase coding and these specific cognitive deficits, and this is a promising area for further research.

## Discussion

**Figure 2:**
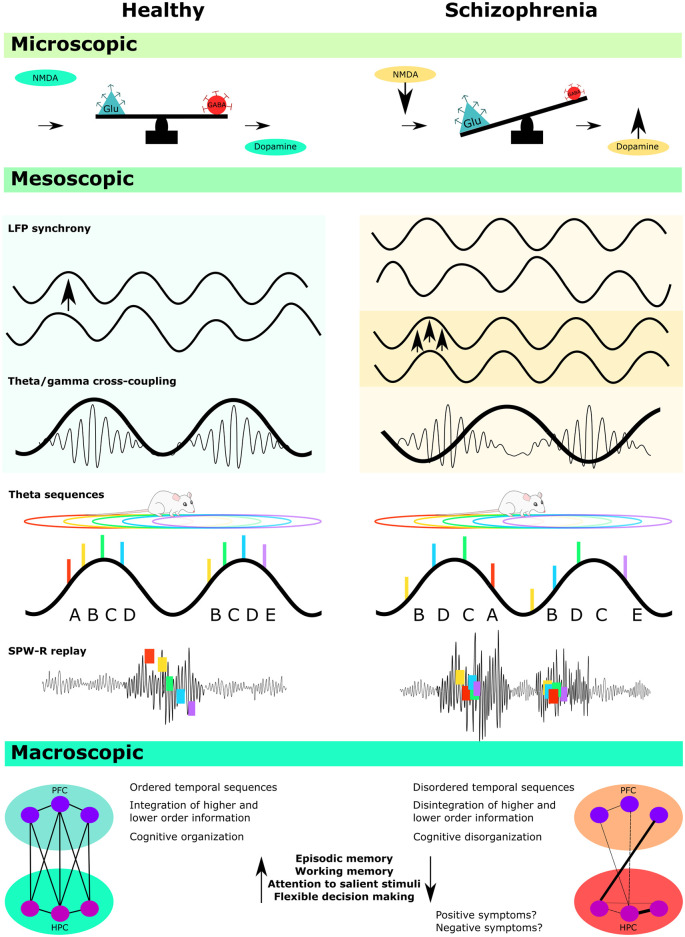
Disorganized oscillatory activity provides the mesoscopic link between microscopic disruptions at the cellular and molecular level, and macroscopic outcomes for impaired cognition in schizophrenia. At the microscopic level, hypofunction at the site of NMDA receptors leads to an imbalance of excitatory/inhibitory regulation in schizophrenia. This in turn is thought to lead to dysregulation of dopamine transmission, with hyperdopaminergic activity predominant in sub-cortical regions. At the mesoscopic level, local field potential (LFP) synchrony is disturbed across several frequency bands, including theta and gamma. This can manifest as a desynchronized activity within and between hippocampal and prefrontal regions, and disturbed theta/gamma cross coupling. A failure to coordinate the spiking of single cells relative to the hippocampal theta rhythm also leads to disordered theta sequences and diminished neural syntax across multiple theta cycles, as well as a loss of structured replay activity during sharp-wave ripples. Finally, at the macroscopic level, these disturbances are thought to contribute to functional dysconnectivity across distributed networks. At the cognitive and behavioral levels, this manifests as diminished performance across a range of tasks.

In summary, we have described how oscillations in neural systems may serve as a scaffold upon which coherence and communication can be achieved within and between brain regions. We have also discussed how disruptions in these oscillatory mechanisms could lead to the kind of disorganized processing and functional disintegration that is observed in schizophrenia, to the degree that it might underlie some of the core features of the disorder, particularly the disruption of episodic memory and planning processes. While dysfunction in a number of different brain regions is likely to occur in schizophrenia, we have chosen to focus on the hippocampus because of its role in encoding sequential information across time and space. The use of animal models has allowed for a detailed examination of the biological mechanisms that might underlie these processes, with current evidence pointing to local GABAergic circuits as a critical component of coordinated spiking activity, as well as network synchrony within and between the hippocampus and PFC. A graphical overview of these disruptions as they occur at the microscopic, mesoscopic, and macroscopic levels is provided in Figure 2.

In particular, we have focussed on phase precession and theta sequences because of their potential to underlie certain types of sequence learning, and have described how a disruption of phase precession, as observed in the MIA model, could result in a fundamental disorganization of sequential information processing. If a similar dysfunction occurs in schizophrenia, it may contribute to several symptoms of cognitive disorganization that have been documented in schizophrenia, such as and impaired episodic and working memory, diminished future planning, thought disorder, and misattributions of agency and control. Taken together with the large body of evidence documenting sequential processing and episodic memory deficits in schizophrenia, these findings suggest that investigating disorganized phase coding in different animal models of the disorder is a promising area for future research.

Correlational evidence linking disturbed oscillatory processes to cognitive dysfunction has been provided across a number of animal models of the disorder, although this work is still in its early stages. In particular, more direct manipulations that target oscillatory activity within specific frequency ranges are still required to confirm that these phenomena are causally linked. Such studies are currently difficult due to the complex nature of the oscillatory activity that occurs across distributed networks, but emerging evidence describing the basic level mechanisms of coordinated network synchrony and phase coding, in addition to technological advances, is likely to open up new pathways for animal research in this domain.

Finally, animal models of the disorder with good construct, face, and predictive validity have the potential to allow for the complex aetiological and developmental processes associated with schizophrenia to be unpacked, including the pathological trajectories that contribute to disorganized oscillatory at critical stages of neural development and maturation. At the present time, however, a number of research questions addressing these issues remain unanswered. Future studies that attempt to attenuate abnormal network synchrony and phase coding disturbances in animal models *via* administration of either antipsychotics or drugs that specifically target dysfunctional inhibitory networks, will help to clarify whether the disorganized oscillatory activity may be a viable target for preclinical interventions, as well as the development of novel treatments.

## Author Contributions

These authors have contributed equally to this work. All authors contributed to the article and approved the submitted version.

## Conflict of Interest

The authors declare that the research was conducted in the absence of any commercial or financial relationships that could be construed as a potential conflict of interest.

## Publisher’s Note

All claims expressed in this article are solely those of the authors and do not necessarily represent those of their affiliated organizations, or those of the publisher, the editors and the reviewers. Any product that may be evaluated in this article, or claim that may be made by its manufacturer, is not guaranteed or endorsed by the publisher.
